# Neural Correlates of Garment Fit and Purchase Intention in the Consumer Decision-Making Process and the Influence of Product Presentation

**DOI:** 10.3389/fnins.2021.609004

**Published:** 2021-08-10

**Authors:** Hesun Erin Kim, Joon Hee Kwon, Jae-Jin Kim

**Affiliations:** ^1^Institute of Behavioral Science in Medicine, Yonsei University College of Medicine, Seoul, South Korea; ^2^Department of Psychiatry, Yonsei University College of Medicine, Seoul, South Korea

**Keywords:** e-commerce, fit satisfaction, purchase intention, consumer decision, product presentation

## Abstract

In today’s competitive e-commerce markets, it is crucial to promote product satisfaction and to quickly identify purchase intention in decision-making consumers. The present investigation examined the relationship between perceived garment fit and purchase intention, together with how product presentation methods (mannequin versus self-model) contribute to decision-making processes of clothing. Thirty-nine female volunteers were scanned using fMRI while performing an online shopping task. In Part 1, univariate analysis was conducted between garment fit and product presentation factors to assess their effects on purchase deliberation. In Part 2, univariate, multivariate pattern, and psychophysiological interaction analyses were carried out to examine the predictive ability of fit evaluation and product presentation on purchase intention. First, garment fit × product presentation interaction effects on purchase deliberation were observed in the frontopolar cortex, superior frontal gyrus, anterior cingulate cortex, and posterior cingulate cortex. Part 2 demonstrated neural signals of the dorsomedial prefrontal cortex, premotor cortex, supplementary motor area, superior parietal lobule, supramarginal gyrus, superior temporal sulcus, fusiform gyrus, and insula to distinguish subsequent purchase intentions. Overall, the findings denote directed exploration, visual and action processing as key neural processes in decision-making that uniquely reflect garment fit and product presentation type during purchase deliberation. Additionally, with respect to the effects of purchase intention on product evaluation, the evidence conveys that mental interactions with products and social cognition are fundamental processes that capture subsequent purchase intention at the product evaluation stage.

## Introduction

Consumers go through complex internal processes before making purchase decisions. As consumers are the backbone of all businesses, understanding the buying behavior is critical. Consumer purchase intention informs businesses about customers and purchase intention is one of the strongest indicators of future purchase decisions (Brown, 2003). Marketplace globalization and a shift toward e-commerce have contributed to highly competitive consumer environments (Wind and Mahajan, 2002). With the phenomenal growth of e-commerce in retail, it has become even more critical to discern consumer’s inclination to purchase early in the decision process. Evident in studies that model early purchase intention prediction in e-commerce settings, the way one interacts with websites and products is meaningfully different before making a purchase decision (Mokryn et al., 2019; Esmeli et al., 2020; Kim et al., 2020).

With the application of neuroscience to the understanding of consumer psychology (Smidts et al., 2014), extensive research has identified how various factors influence the consumer behavior and elucidating their neural basis has opened doors to many possibilities. For example, Knutson et al. (2007) presented evidence of the involvement of the nucleus accumbens, mesial prefrontal cortex, and insula in predicting decision outcomes prior to making the decision. More specifically, this instrumental study had subjects engage in a shopping task, where they viewed the product (“preference period”) and the price (“price period”), and then chose to buy or not. The comparison of time courses of several brain regions indicated that the nucleus accumbens and mesial prefrontal cortex were activated during the preference period and price period, respectively, when subjects chose to buy, whereas insula activity was increased at the price period when they chose not to buy. Another study reported a role of the superior frontal gyrus (SFG) and occipital gyrus in decoding product choices (Van der Laan et al., 2012). These reports provided undoubtedly compelling evidence that consumers’ purchase intentions can be predicted prior to the decision stage. Yet, our knowledge on these regarding purchasing clothing in an e-commerce setting is still insufficient.

When it comes to the fashion industry, there are numerous factors that influence the overall fit satisfaction of apparels, such as color, fabric texture, comfort, body type, function, personality, garment fit, and how products are presented to customers (Radeloff, 1991; Chattaraman and Rudd, 2006; Pisut and Connell, 2007; Tiggemann and Lacey, 2009; Kim and Damhorst, 2010). In general, the overall fit satisfaction of clothing is evaluated through both visual and tactile information, and thus physical evaluation is important in the decision to purchase clothes (Ashdown and DeLong, 1995; Le Pechoux and Ghosh, 2002). Despite the growth of digital sales within the fashion industry, the risk of dissatisfaction with a choice without physical evaluation remains one of the downsides of e-commerce shopping. Subsequently, the importance of visual information over tactile ones has emphasized in an e-commerce setting. In particular, the perception of physical or esthetic fit of the garment is among the most critical attributes that shape purchasing behavior and attitudes in clothing (Alexander et al., 2005; Hwang et al., 2016). Another important factor is product presentation. Images of products on models and mannequins attract more attention from consumers than zoomed images of items and enhance purchase intention (Boardman and McCormick, 2019). Providing consumers with dynamic imagery, such as virtual try-on, have been shown to reduce the gap between online and offline shopping experience and mitigates the perceived risk (Kim and Forsythe, 2008). Even though these two factors, garment fit and product presentation, are important for purchase deliberation in e-commerce shopping, the brain mechanism of the process remains unexplained.

Several probable brain regions can be proposed for the neural basis of factors influencing consumer behaviors toward fashion products. For example, different methods of product presentation have not only indicated greater engagements of neural regions related to visual processing, mental imagery, and reward processing, but also have shown the effectiveness in altering purchasing behavior (Jai et al., 2014). Reward-related regions including the ventral striatum and ventromedial prefrontal cortex (vmPFC) may be important because they are involved in one’s desires for stimuli (Pool et al., 2016). Since sensory experiences are essential in consumer behavior (Krishna, 2012), the superior parietal lobule (SPL), supramarginal gyrus (SMG), and inferior frontal gyrus (IFG) can be recruited. The SPL is regarded as a key contributor in mental imagery (Gourtzelidis et al., 2005) and sensory aspects of decision-making (Zhou and Freedman, 2019). There is evidence that a robust intention-to-purchase is induced through mental imagery by the SPL (Liu et al., 2018). The SMG has abundant mirror neurons (Chong et al., 2008) that integrates reward and other factors for action-reward associations (Vickery and Jiang, 2009). The IFG has also been implicated in the mental imagery network and mirror neuron system (Rizzolatti and Craighero, 2004). These mirror neuron-rich areas, often implicated in mental imagery, may possibly predict the presence of purchase intention during product evaluation. Purchasing processes for apparel also involve both personal and social components (Tiggemann and Lacey, 2009; Shin and Damhorst, 2018). Since consumers formulate fit satisfaction from both their own and others’ perspectives, the engagement of self-referential processing by the dorsomedial prefrontal cortex (dmPFC) (D’Argembeau et al., 2007) and mentalization by the temporoparietal junction (TPJ) (Saxe and Kanwisher, 2003) and superior temporal sulcus (STS) (Deen et al., 2015) may be essential in the consumer decision-making process of fashion products.

Despite the intricate workings of the factors that influence the consumer process for fashion products, neural underpinnings of these factors have not yet been elucidated. This study aimed to understand the neural basis underlying garment fit, purchase intention, as well as mannequin and self-model product presentation methods through two parts. Part 1 investigated the neural effects of garment fit and presentation type on purchase deliberation. Here, we hypothesized that the items with garment fit presented on self-models would particularly engage the reward network including the ventral striatum and vmPFC during deliberation of purchase decision. Part 2 sought to identify brain regions that predict purchase intention under the hypothesis that the way decision makers interact with products during fit evaluation would uniquely capture the subjective intention-to-purchase and product presentation types, and this way would be reflected in neural areas associated with mental imagery, such as the SPL, SMG, and IFG, and social cognition, such as the dmPFC, TPJ, and STS.

## Materials and Methods

### Participants

A total of 39 healthy female participants between the ages of 20 and 29 years were recruited via online advertisement (age, 23.5 ± 2.1 years; education, 16.3 ± 1.9 years). Exclusion criteria included left-handedness, pregnancy, and neurological or psychiatric diseases. All participants were provided informed written consent prior to partaking in the study, and the study was approved by the Institutional Review Board of Yonsei University Severance Hospital and carried out in accordance with the Declaration of Helsinki.

### Experimental Procedure

**FIGURE 1:**
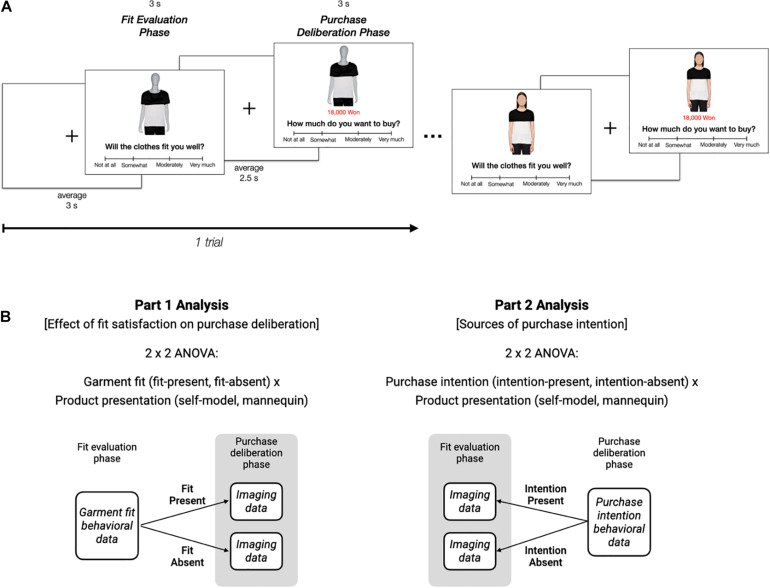
Experimental and analysis design. **(A)** Each participant was presented with an apparel either on a mannequin or on a model with the participant’s face superimposed (self-model). First, participants were instructed to disclose their garment fit rating (fit evaluation phase). Next, they were asked to indicate how much they would like to purchase the clothing (purchase deliberation phase). **(B)** In Part 1, trials were separated based on participant’s behavioral responses of garment fit-present and fit-absent, and then neuroimaging data of the subsequent purchase deliberation phase were analyzed. In Part 2, trials were separated based on participant’s behavioral responses of purchase intention-present and purchase intention-absent, and then neuroimaging data of the previous fit evaluation phase were analyzed.

During the fMRI scanning session, participants engaged in an apparel-purchasing task. Task stimuli were prepared before the scanning session. A set of 42 articles of top-only clothing was selected from various online shopping websites. To maximize generalizability, selected items consisted of short-sleeved shirts, long-sleeved shirts, and sweaters. Using Adobe Photoshop (Creative Suite 6, Adobe, United States), all products were traced for manipulation and all brand labels (or any indication of such) were erased to eliminate the brand effects. Then, the manipulation-ready products were transposed onto a generic mannequin and a female model. The images of both the mannequin and female model were framed to show the bodies from the top of the head to above the knees and from shoulder to shoulder. Participants completed an online survey to approximate an appropriate market price for each item of clothing a few days prior to the fMRI scan and provided a picture of their own face. Self-model images of each participant were produced by superimposing participants’ faces onto the female model’s body to make each shopper feel like she was wearing the clothes (Figure 1A).

The task sequence included two runs and 42 trials in each run of approximately 7 min 52 s. In the first run, 42 different articles of clothing were used in the form of 21 self-model and 21 mannequin images, with one per trial. The same 42 clothes were used interchangeably between self-model and mannequin images in the second run. Each trial consisted of two phases of 3 s, representing fit evaluation and purchase deliberation, to emulate the consumer decision-making process. In the fit evaluation phase, one of the prepared superimposed images of either the mannequin or the participant herself wearing an apparel was presented. At the bottom of the image appeared the question, ‘‘Will the clothes fit you well?’’ Together with this, a four-ticked Likert scale appeared with scores of 0: ‘‘not at all,’’ 1: ‘‘somewhat,’’ 2: ‘‘moderately,’’ 3: ‘‘very much.’’ Participants were instructed to indicate the level of garment fit by pressing a button, and the response was referred to as the fitness score. In the purchase deliberation phase, the identical image from the fit evaluation phase was displayed with the price along with the question, ‘‘How much do you want to buy?’’ Also displayed was the identical Likert scale. Participants’ responses were referred to as the intention-to-purchase score. In order to limit the influence of price information as the standards for the price of apparels vary from person to person, the prices were adjusted and presented in reference to the individual’s pre-reported price point. On average, the prices the participants saw were approximately discounted by 12%, where the highest markdown was by 50% and the highest markup was by 130% of the pre-reported price points. To encourage serious participation in the task, participants were informed that they could purchase one product of high intention-to-purchase at the end of the scanning session. The intervals were jittered with an average of 2.5 s (range: 1–4.5 s) between the phases and 3 s (range: 2–6 s) between the trials. The schedule of events and jitters were obtained using Optseq2.^1^

### Imaging Data Acquisition and Preprocessing

All functional scanning was performed on 3.0 Tesla MRI scanner (Ingena 3.0T CX, Philips Healthcare, Best, Netherlands) with a 32-channel head coil. For each participant, echo-planar imaging scans were acquired with the following parameters: repetition time = 2,000 ms, echo time = 30 ms, flip angle = 90°, number of acquisitions = 235, number of slices = 31, slice thickness = 3 mm with 1 mm interstitial gap, and matrix size = 80 × 80. A high-resolution T1-weighted structural scan was also obtained from each participant using a 3D gradient echo (matrix size = 256 × 256, number of slices = 180, and slice thickness = 1 mm) after the functional scan.

All images were preprocessed using Statistic Parametric Mapping 12 (SPM 12^2^). To allow for the stabilization of magnetization, the first five scans were discarded. The remaining images were corrected for slice-timing, realigned to correct for head-motion, and co-registered on the individual T1-weighted image. Then, the T1-image was spatially normalized to the MNI template and the resulting transformation matrices were applied to the co-registered functional images. The normalized images were smoothed with a Gaussian kernel of 6-mm full-width at half-maximum (FWHM).

### Behavioral Data Analysis

#### Part 1: Effects of Fit Satisfaction

To assess the effects of garment fit and product presentation on purchase intention in the purchase deliberation phase, differences in the intention-to-purchase scores and reaction times (RTs) among the categories were examined using repeated-measures ANOVA. To determine garment fit, the participants’ responses during the fit evaluation phase were separated according to each participant’s fitness rating obtained during in-scan behavior; a score of 0 was deemed as having no garment fit (fit-absent) and scores of 1, 2, and 3 were deemed as having at least some garment fit (fit-present). The trials during the purchase deliberation phase were then labeled depending on the product presentation conditions (self-model and mannequin), thereby creating four categories (garment fit × product presentation). *Post hoc* analysis of any significant main effects or interaction effects was then performed using paired *t*-tests.

#### Part 2: Sources of Purchase Intention

To assess the predictive ability of garment fit and product presentation on the subsequent purchase intention, differences in the fitness scores and RTs among the categories were assessed using repeated-measures ANOVA.

To determine purchase intention, participants’ responses during the purchase deliberation phase were categorized into intention-absent and intention-present for an intention-to-purchase score of 0 corresponding to having no intention to buy and scores of 1, 2, and 3 corresponding to having some purchase intention, respectively. The trials during the fit evaluation phase were then labeled depending on the product presentation conditions (self-model and mannequin), thereby creating four categories (purchase intention × product presentation). *Post hoc* analysis was carried using paired *t*-test. All analysis of behavior data was computed using SPSS 25.0 (SPSS Inc., Chicago, IL, United States).

### Mass Univariate Imaging Analysis

Once preprocessed, imaging data was analyzed using a general linear model (GLM) at a single-subject level. The preprocessed images were separated into eight categories; the images obtained during the purchase deliberation phase were divided according to garment fit (fit-present and fit-absent) and product presentation (self-model and mannequin), and those obtained during the fit evaluation phase were divided according to purchase intention (intention-present and intention-absent) and product presentation (self-model and mannequin). Then, the BOLD signals for each category were modeled at the onset of each stimulus for the duration of RT (“variable epoch”) to factor out the effects of RTs (Grinband et al., 2008), and the signals were convolved with the Hemodynamic Response Function. Additional six rigid head motion parameters acquired during preprocessing were included as regressors of no interest, and high-pass filter was applied at 128 Hz to reduce low-frequency drift and physiological noise. Fixation was not modeled. To investigate the effects of garment fit and product presentation on purchase deliberation (Part 1) and find the neural sources predicting purchase intention (Part 2), the contrast images of garment fit × product presentation at the purchase deliberation phase and those of purchase intention × product presentation at the fit evaluation phase were entered into the flexible factorial model at the group level analysis (Figure 1B). Statistical inferences were set at a threshold of *P*_*UNC*_ < 0.001. The multiple comparison problem was addressed using family-wise error correction at *P*_*FWE*_ < 0.05 at the cluster level.

### Multivariate Pattern Analysis

For Part 2, as a complementary analysis to the mass univariate analysis, we performed multivariate pattern analysis (MVPA) for whole brain searchlight to demonstrate the brain correlates that are predictive of purchase intentions from the fit evaluation phase. The decoding toolbox optimized for SPM was used to decode brain regions showing unique neural patterns reflecting subsequent intention-to-purchase. As recommended by Hebart et al. (2015), non-normalized and non-smoothed preprocessed data were used. Similar to the setup used in “Mass univariate imaging analysis,” the GLM was estimated for the purchase deliberation phase of each trial according to garment fit and product presentation, and the fit evaluation phase of each trial according to purchase intention behavior and product presentation as a separate and single regressor, creating a maximum of 84 separate beta images per run for every participant. The regressors were modeled at the onset of each stimulus with RT as duration, and the six rigid head motions were included as regressors of no interest.

The regressors of the fit evaluation phase were labeled by the intention-to-purchase category, and data were split into half by the first and second run. Next, we took a cross-validated pattern correlation approach (Haxby et al., 2001). A spherical searchlight with a radius of 10 mm was defined for every voxel in a brain mask. In each voxel, the mean signal was subtracted across all categories. The neural activity of voxels within each searchlight was averaged across trials and categories. To identify the spatial pattern unique to each category, we compared the correlations between the response pattern to one category in the first data set and the pattern of the same category in the second data set, namely within category correlation. Then, we compared the correlations between the two categories, known as between-category correlation. Results of analysis were reported in terms of the area under the receiver operating characteristics curve, a graphical plot that represents graded decision values and better addresses any possible classification bias. The chance level was set at 50%.

The resulting map of the decoded brain was normalized and smoothed with a Gaussian kernel of 6-mm FWHM. Then, the map was entered into one-tailed one-sample *t*-tests to examine the unique neural patterns predictive of subject’s subsequent intention-to-purchase. As MVPA has been known to increase sensitivity of cognitive states (Norman et al., 2006), a stricter threshold was set at uncorrected *P* < 0.0001 voxel-wise and *P*_*FWE*_ < 0.05 cluster-wise correction.

### Psychophysiological Interaction Analysis

Computation of the functional coupling between one of the chosen seeds and the entire brain in response to a task (Friston et al., 1997) was conducted using the CONN toolbox optimized for SPM12 (Whitfield-Gabrieli and Nieto-Castanon, 2012). The setup of the model was as described above in the mass univariate GLM. Any hypothesized region (SPL, SMG, IFG, dmPFC, TPJ, and STS) observed in our mass univariate analysis of purchase intention was defined as seed ROIs. Each spherical ROI mask with 5 mm radius around the peak coordinate was created. The interaction regressor between time series of each seed region (physiological term) and the task conditions (psychological term) was modeled for the difference between intention-present and intention-absent categories during the fit evaluation phase. The interaction regressor produced the connectivity modulation for each category across every voxel. Individual seed-to-voxel connectivity maps were entered into paired *t*-tests to assess between-category effects. The statistical threshold was set as described in the mass univariate analysis.

## Results

### Effects of Fit Satisfaction

#### Behavioral Results

The summary of behavioral data is presented in Table 1. In the fit evaluation phase, an average of 35 versus 65% of the trials were identified as fit-absent and fit-present, respectively. Repeated-measures ANOVA of the intention-to-purchase scores revealed a main effect of garment fit (*F*_1,38_ = 334.87, *P* < 0.001), but no main effect of product presentation or interaction effect of garment fit × product presentation. *Post hoc* analysis showed that the intention-to-purchase scores for fit-present items were significantly higher than the scores for fit-absent products (*t*_38_ = 18.37, *P* < 0.001). Analysis of RTs showed main effects of garment fit (*F*_1,38_ = 102.96, *P* < 0.001) and product presentation (*F*_1,38_ = 8.14, *P* = 0.007), but no interaction effect. *Post hoc* analyses indicated that RTs were significantly longer for fit-present than for fit-absent clothing (*t*_38_ = 10.43, *P* < 0.001) and showed shorter RTs toward self-models than toward mannequins (*t*_38_ = −4.94, *P* < 0.001).

**TABLE 1:** Summary statistics (mean ± SD) of behavioral data.

Part 1	Self		Mannequin	
	Fit-present	Fit-absent	Fit-present	Fit-absent
***Data obtained at purchase deliberation phase***				
Intention-to-purchase score	1.39 ± 0.46	0.10 ± 0.14	1.32 ± 0.37	0.10 ± 0.16
Intention-to-purchase RT (ms)	1322.41 ± 236.14	977.56 ± 223.13	1375.96 ± 224.87	1016.24 ± 238.78

**Part 2**	**Self**		**Mannequin**	
	**Intention-present**	**Intention-absent**	**Intention-present**	**Intention-absent**

***Data obtained at fit evaluation phase***				
Fitness score	1.64 ± 0.36	0.34 ± 0.23	1.77 ± 0.37	0.54 ± 0.42
Fitness RT (ms)	1622.98 ± 240.94	1426.22 ± 257.74	1598.16 ± 261.43	1428.69 ± 256.22

*RT, reaction time.*

#### Results From the Mass Univariate Analysis

**FIGURE 2:**
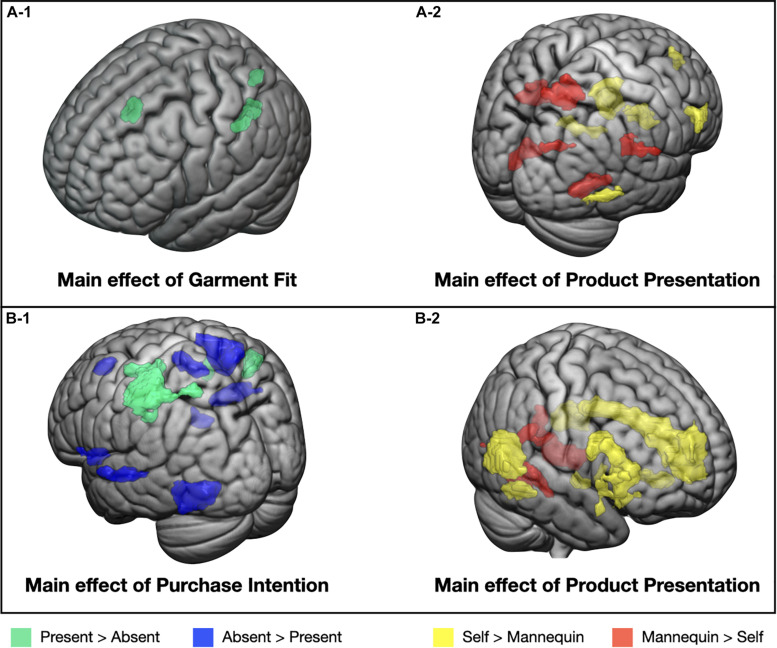
An illustration of significant main effects from the univariate analysis. Significant neural clusters were illustrated for the main effects of garment fit **(A-1)** and product presentation **(A-2)** in Part 1 and for the main effects of purchase intention **(B-1)** and product presentation **(B-2)** in Part 2.

Table 2 presents the significant brain regions showing significant main effects of garment fit and product presentation and interaction effect between the two factors at the purchase deliberation phase. The main effect of garment fit was observed in the bilateral supplementary motor area (SMA), left SPL, and left SMG (Figure 2A-1). All of these regions activated more for fit-present than fit-absent conditions. The main effect of product presentation was seen in various cortical and subcortical regions (Figure 2A-2). *Post hoc* tests demonstrated that the bilateral dmPFC, right dorsolateral prefrontal cortex (dlPFC), right inferior temporal gyrus, bilateral midcingulate cortex, left hippocampus, and bilateral caudate showed greater activity for self-models than for mannequins, whereas the right SMG, bilateral precuneus, left STS, and bilateral fusiform gyrus exhibited greater activity for mannequins than for self-models.

**TABLE 2:** Significant brain regions in the mass univariate analysis showing the effects of garment fit and product presentation type during the purchase deliberation phase.

Region	HEM	Cluster size	*F*	MNI coordinates			*Post hoc*
				*x*	*y*	*z*	
***Main effect of garment fit***							
SMA	B	118	15.10	4	16	46	Present > absent
SPL	L	93	20.83	−12	−70	46	Present > absent
Supramarginal gyrus	L	323	23.57	−44	−42	40	Present > absent
***Main effect of product presentation***							
dmPFC	B	101	25.30	2	58	22	Self > mannequin
dlPFC	R	230	20.10	44	32	8	Self > mannequin
Inferior temporal gyrus	R	168	29.92	52	−62	−8	Self > mannequin
MCC	B	123	19.93	0	−6	34	Self > mannequin
Hippocampus	L	220	26.96	−30	−14	−10	Self > mannequin
Caudate	L	465	58.17	−10	8	2	Self > mannequin
	R	306	19.67	10	8	6	Self > mannequin
Supramarginal gyrus	R	196	23.11	46	−30	18	Mannequin > self
Precuneus	B	410	27.35	6	−54	48	Mannequin > self
	R	119	12.40	16	−54	16	Mannequin > self
STS	L	480	37.95	−46	−28	10	Mannequin > self
Fusiform gyrus	L	255	25.11	−26	−48	−14	Mannequin > self
	R	396	27.41	34	−46	−10	Mannequin > self
***Interaction effect of garment fit × *product presentation****							
Frontopolar cortex	R	212	30.31	26	50	16	Figure 3A
SFG	R	108	20.50	24	12	52	Figure 3B
ACC	B	1,389	58.34	−4	32	14	Figure 3C
PCC	B	94	16.29	−2	−38	42	Figure 3D

*HEM, hemisphere; MNI, Montreal Neurological Institute; B, bilateral; L, left; R, right; SMA, supplementary motor area; SPL, superior parietal lobule; dmPFC, dorsomedial prefrontal cortex; dlPFC, dorsolateral prefrontal cortex; MCC, middle cingulate cortex; STS, superior temporal sulcus; SFG, superior frontal gyrus; ACC, anterior cingulate cortex; PCC, posterior cingulate cortex.*

**FIGURE 3:**
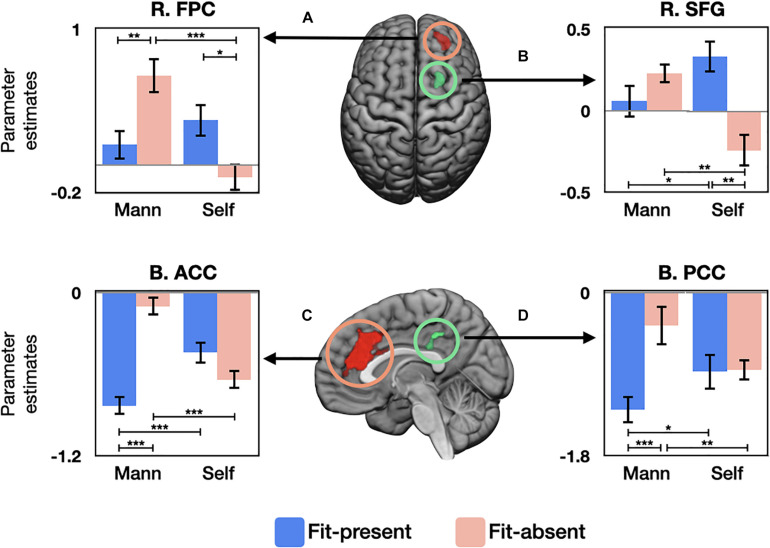
Graphs of parameter estimates of neural regions. Panels **(A–D)** showing interaction effects between garment fit and product presentation type during purchase deliberation. Mann, mannequin; R., right; L., left; B., bilateral; FPC, frontopolar cortex; SFG, superior frontal gyrus; ACC, anterior cingulate cortex; PCC, posterior cingulate cortex. *P < 0.05, **P < 0.01, ***P < 0.001.

The interaction effect of garment fit × product presentation was evident in the right frontopolar cortex (FPC), right SFG, bilateral anterior cingulate cortex (ACC), and bilateral posterior cingulate cortex (PCC). Results from *post hoc* analysis are presented in Figure 3. In the right FPC (Figure 3A), its activity was significant higher for fit-present/self-models than for fit-absent/self-models (*t*_38_ = 2.07, *P* = 0.046), lower for fit-present/mannequins than for fit-absent/mannequins (*t*_38_ = −3.24, *P* = 0.003), and lower for fit-absent/self-models than for fit-absent/mannequins (*t*_38_ = −4.49, *P* < 0.001). In the right SFG (Figure 3B), fit-present items prompted significantly higher activation for self-models than for mannequins (*t*_38_ = 2.33, *P* = 0.025), whereas fit-absent items generated significantly lower activity for self-models than for mannequins (*t*_38_ = −3.24, *P* = 0.002). There was no significant difference in activation between fit-present and fit-absent within products displayed on mannequins (*t*_38_ = −1.05, *P* = 0.302), but the SFG activation was greater for fit-present than fit-absent items within self-models (*t*_38_ = 3.72, *P* = 0.001). In the bilateral ACC (Figure 3C), the activation was greater for fit-present/self-models than for fit-present/mannequins (*t*_38_ = 4.26, *P* < 0.001), whereas its activity was lower for fit-absent/self-models than fit-absent/mannequins (*t*_38_ = −5.16, *P* < 0.001). Fit-present items induced significantly lower activity than fit-absent items (*t*_38_ = −4.26, *P* < 0.001) within mannequins, but no significant difference was observed between fit-present and fit-absent items within self-models (*t*_38_ = 1.57, *P* = 0.125). In the bilateral PCC (Figure 3D), the activity was significantly higher for fit-present/self-models than for fit-present/mannequins (*t*_38_ = 1.07, *P* = 0.045), whereas it was significantly lower for fit-absent/self-models than for fit-absent/mannequins (*t*_38_ = −2.86, *P* = 0.007). Within mannequins, the activity of fit-present condition was lower than that of fit-absent condition (*t*_38_ = −4.82, *P* < 0.001), but no difference was observed between fit-present than fit-absent products within self-models (*t*_38_ = −0.08, *P* = 0.934).

### Sources of Purchase Intention

#### Behavioral Results

The summary of behavioral data is also shown in Table 1. In the purchase deliberation phase, an average of 48 versus 52% of the trials were identified as intention-absent and intention-present, respectively. Analysis of the fitness scores based on the presence of intention-to-purchase and product presentation revealed significant main effects of purchase intention (*F*_1,38_ = 491.84, *P* < 0.001) and product presentation (*F*_1,38_ = 10.58, *P* = 0.002), and a moderately significant interaction effect between the two factors (*F*_1,38_ = 3.03, *P* = 0.09). The fitness scores were significantly higher for garments that participants later identified as having intention-to-purchase than those without intention-to-purchase (*t*_38_ = 23.81, *P* < 0.001) and toward clothing displayed on a mannequin than on the participants themselves (*t*_38_ = 3.25, *P* = 0.002). Specifically, the fitness scores were higher for garments displayed on mannequins that were later identified as intending to purchase than for garments displayed on mannequins that participants did not intend to purchase (*t*_38_ = 20.12, *P* < 0.001). Also, the fitness scores were higher for intention-present clothing displayed on mannequins in comparison to intention-present clothing displayed on self-models (*t*_38_ = 2.29, *P* = 0.027). In contrast, the scores were higher for intention-present garments displayed on self-models than for intention-absent garments displayed on self-models (*t*_38_ = 21.47, *P* < 0.001). Regarding intention-absent products, the scores were higher when items were presented on mannequins than on self-models (*t*_38_ = 3.74, *P* = 0.001). The RTs showed a main effect of purchase intention (*F*_1,38_ = 50.63, *P* < 0.001), but displayed no main effect of product presentation and interaction effect. *Post hoc* analysis indicated that the RTs for intention-present apparels were significantly longer than for intention-absent clothing (*t*_38_ = 7.00, *P* < 0.001).

#### Results From the Mass Univariate Analysis

Table 3 presents brain regions showing significant main effects of purchase intention and product presentation and interaction effect between the two factors during the fit evaluation phase. The main effect of purchase intention was observed in various cortical regions (Figure 2B-1). *Post hoc* analyses showed that the right premotor cortex, left SPL, and right SMG were more activated for intention-present than for intention-absent clothing, whereas the bilateral dmPFC, right SMA, right SPL, left STS, left fusiform gyrus, left anterior insula, right posterior insula, and right dorsal striatum were more activated for intention-absent products than for intention-present products. Several clusters also showed the main effect of product presentation (Figure 2B-2). Specifically, the bilateral dmPFC/rostral ACC, right posterior orbitofrontal cortex (pOFC), bilateral TPJ, right fusiform gyrus, bilateral anterior insula, right nucleus accumbens, and midbrain showed greater activation toward products displayed on self-models than on mannequins, whereas the bilateral fusiform gyrus activated more for products displayed on mannequins than on self-models. No brain region showed the significant interaction effect.

**TABLE 3:** Significant brain regions in the mass univariate analysis showing the prediction ability of the imaging data during the fit evaluation phase for purchase intention and product presentation type.

Region	HEM	Cluster size	*F*	MNI coordinates			*Post hoc*
				*x*	*y*	*z*	
***Main effect of purchase intention***							
Premotor cortex	R	117	30.00	50	4	28	Present > absent
SPL	L	1,382	34.53	−32	−24	58	Present > absent
Supramarginal gyrus	R	373	28.96	46	−40	48	Present > absent
dmPFC	B	141	31.90	−6	48	32	Absent > present
SMA	R	274	39.54	8	−26	50	Absent > present
SPL	R	1,529	18.69	24	−46	64	Absent > present
STS	L	270	21.23	−56	−6	−6	Absent > present
Fusiform gyrus	L	905	26.81	−28	−58	−14	Absent > present
Anterior insula	L	178	26.77	−36	20	−10	Absent > present
Posterior insula	R	386	37.58	46	−22	20	Absent > present
Dorsal striatum	R	136	28.06	32	−10	−2	Absent > present
***Main effect of product presentation***							
dmPFC/rACC	B	2,408	42.22	4	56	20	Self > mannequin
pOFC	R	205	31.41	26	20	−18	Self > mannequin
Temporoparietal junction	L	512	36.13	−54	−66	6	Self > mannequin
	R	825	60.84	52	−58	6	Self > mannequin
Fusiform gyrus	R	184	79.14	44	−46	−20	Self > mannequin
Anterior insula	L	213	32.53	−28	16	−18	Self > mannequin
	R	1,047	35.49	36	26	0	Self > mannequin
Nucleus accumbens	R	165	24.57	4	6	−8	Self > mannequin
Midbrain		122	18.50	−6	−18	−14	Self > mannequin
Fusiform gyrus	L	582	44.29	−28	−56	−14	Mannequin > self
	R	764	36.48	34	−44	−10	Mannequin > self
***Interaction effect of purchase intention × product presentation***							
None							

*HEM, hemisphere; MNI, Montreal Neurological Institute; B, bilateral; L, left; R, right; SPL, superior parietal lobule; dmPFC, dorsomedial prefrontal cortex; SMA, supplementary motor area; STS, superior temporal sulcus; rACC, rostral anterior cingulate cortex; pOFC, posterior orbitofrontal cortex.*

#### Results From the Multivariate Pattern Analysis

**FIGURE 4:**
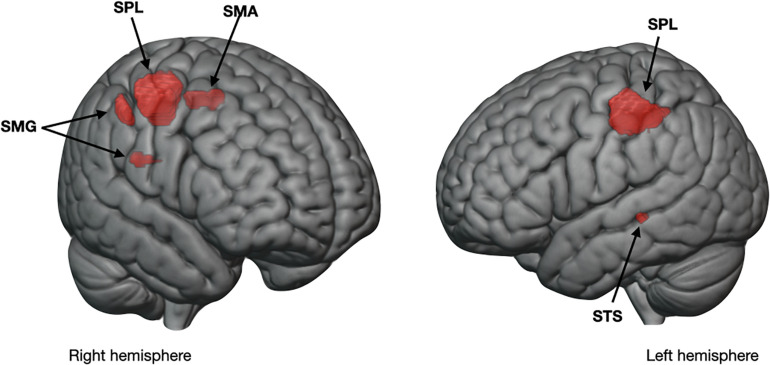
Overlapping neural areas of purchase intention. An overlay of significant clusters obtained from mass univariate analysis showing a main effect of purchase intention and from multivariate pattern analysis demonstrating classification accuracy above the chance level (50%). SPL, superior parietal lobule; SMA, supplementary motor area; SMG, supramarginal gyrus; STS, superior temporal sulcus.

Table 4 lists neural regions where patterns of activity correctly identified the presence of purchase intention above the chance level of 50% during participants’ fit evaluation. In the order of highest to lowest classification accuracy, identified regions were the bilateral SPL, right fusiform gyrus, right SMG, left middle temporal gyrus, right thalamus, right middle temporal gyrus, right pOFC, left dmPFC, right STS, left superior temporal gyrus, and right premotor cortex. Furthermore, as exploratory MVPA was performed to confirm the mass univariate results showing activational differences between the subsequent presence and absence of purchase intention, Figure 4 overlays the common regions from mass univariate and multivariate pattern analyses. Common regions include the SMA, SPL, and SMG in the right hemisphere, as well as the SPL and STS in the left hemisphere.

**TABLE 4:** Multivariate pattern analysis results of purchase intention during the fit evaluation phase in the order of highest to lowest classification accuracy.

Region	HEM	Cluster size	*T*	MNI coordinates			Classification accuracy %
				*x*	*y*	*z*	
***Purchase intention (present versus absent)***							
SPL	R	3,219	17.37	42	−24	54	83.1
	L	3,973	9.41	−38	−24	54	80.3
Fusiform gyrus	R	185	5.87	24	−54	−22	70.0
Supramarginal gyrus	R	325	6.11	52	−22	24	68.8
Middle temporal gyrus	L	130	6.12	−52	−34	−2	67.9
Thalamus	R	245	6.52	18	−10	−2	67.3
Middle temporal gyrus	R	29	4.79	58	−34	−6	65.4
pOFC	R	31	4.49	34	36	−4	64.4
dmPFC	L	102	4.48	−14	48	42	62.2
STS	R	36	4.47	66	−22	−12	61.5
Superior temporal gyrus	L	43	4.84	−56	14	−6	58.5
Premotor cortex	R	160	6.35	66	8	28	53.1

*Results are reported at voxel-level P_UNC_ < 0.0001 and cluster-level P_FWE_ < 0.05. HEM, hemisphere; MNI, Montreal Neurological Institute; R, right; L, left; B, bilateral; SPL, superior parietal lobule; pOFC, posterior orbitofrontal cortex; dmPFC, dorsomedial prefrontal cortex; STS, superior temporal sulcus.*

#### Results From the PPI Analysis

**FIGURE 5:**
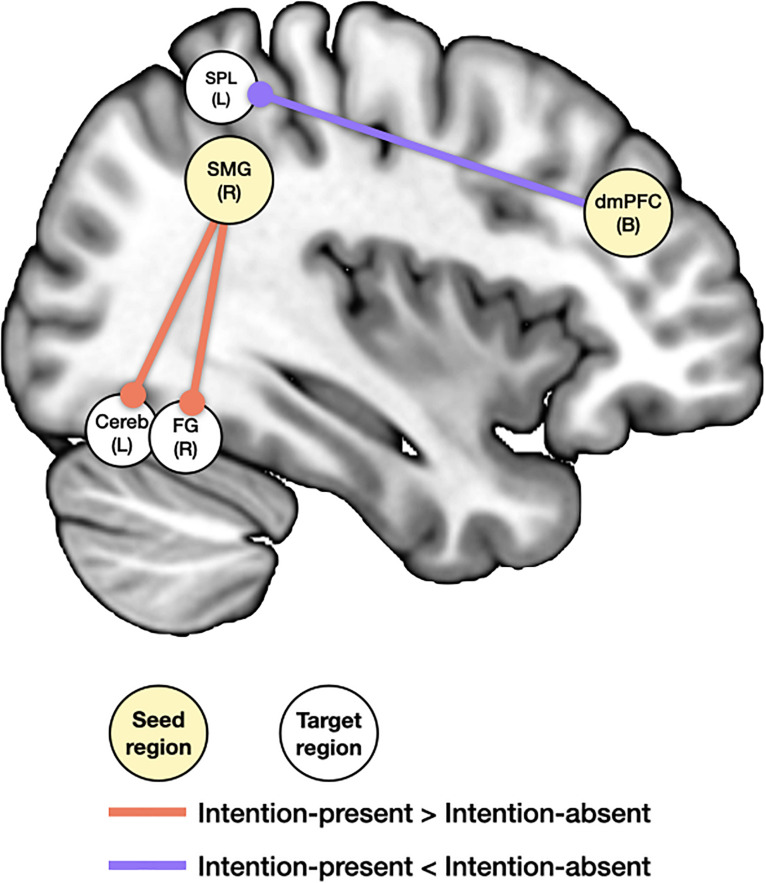
An illustration of psychophysiological interaction effects between purchase intention and product presentation type. The coordinates of seed regions were obtained from peak clusters of mass univariate analysis. L, left; R, right; B, bilateral; SMG, supramarginal gyrus; FG, fusiform gyrus; Cereb, cerebellum; dmPFC, dorsomedial prefrontal cortex; SPL, superior parietal lobule.

Figure 5 presents the results from the psychophysiological interaction (PPI) analysis. Among the clusters showing the main effect of purchase intention in the mass univariate analysis, the bilateral dmPFC, bilateral SPL, right SMG, and left STS were regarded as the hypothesized regions for this analysis. Thus, these regions were selected as the seed ROIs. Significant functional connections in the contrast of intention-present > intention-absent were found in the right fusiform gyrus and left cerebellum with the right SMG. Significant functional connections in the contrast of intention-present < intention-absent were found in the left SPL with the bilateral dmPFC. No other functional connections were significant with the SPL or STS.

## Discussion

The present study aimed to elucidate the neural effects of garment fit and purchase intention on the decision-making process, together with how product presentation influences the relationship. Specifically, we studied how garment fit and product presentation are demonstrated while considering buying a product, then examined the neural signals of fit evaluation period reflecting subsequent purchase intention. Prediction of subsequent purchase intention was further complemented by exploratory MVPA and PPI analyses.

### Effects of Fit Satisfaction

As expected, the intention-to-purchase scores were higher for apparel with garment fit than fit-absent products, but there was no difference in the scores between the presentation types. Participants took longer to disclose purchase intention for fit-present items and toward mannequins than their respective counterparts, suggesting a sense of hesitation when making decisions for items with garment fit, which is an inherent problem of e-commerce shopping. Yet, self-models possibly offer a way to mitigate the perceived risk.

In the garment fit effect, the SMA, SPL, and SMG activated more for fit-present than fit-absent products during purchase deliberation. The SMA is considered to contribute to motor-related activities, cognitive control, sensorimotor representation, and complex motor planning, as well as action, time, and spatial sequence processing (Cona and Semenza, 2017). The SPL has been implicated in sensory aspects of decision-making (Zhou and Freedman, 2019), mental imagery, and mental rotation (Vingerhoets et al., 2002), especially in terms of body part imagery (Overney et al., 2005). In addition, with dense population of mirror neurons, the SMG is thought to integrate information for action-reward associations. These garment fit-related activations may indicate more involvement of mental imagery and perception coupling or reflection when making purchase decisions for clothing with fit satisfaction.

The product presentation effect during purchase deliberation seems to emphasize the involvement of a number of regions related to self-projection. Among the regions where activity increased more in self-models than in mannequins, the dmPFC is a key player for self-referential processing and mentalization (D’Argembeau et al., 2007), while the dlPFC contributes to self-initiated elaborative encoding process (Hawco et al., 2013). It has been reported that the inferior temporal gyrus, hippocampus, and caudate are also involved in self-projection (Kurczek et al., 2015; Herold et al., 2016; Vatansever et al., 2018). On the other hand, mannequins recruited self-related imagery more than self-models through activated structures like the SMG and precuneus (Cona and Semenza, 2017; Grol et al., 2017). Interestingly, the fusiform gyrus and STS, which are activated when viewing a face directly (Pageler et al., 2003), showed increased activity in mannequins than in self-models, which may be a compensatory action for the formation of stronger self-related imagery in mannequins.

In the current study, the interaction effect is of greater interest in light of our hypothesis regarding the brain regions involved in the processing of the items with garment fit presented on self-models. Several regions exhibited interaction effects between garment fit and product presentation. The FPC is a key structure for higher order cognitive functions, including strategy (Domenech and Koechlin, 2015), tracking options (Boorman et al., 2009), and exploration in the explore-exploit dilemma—specifically, directed exploration in uncertain environments (Daw et al., 2006). *Post hoc* tests showed higher activity for fit-present/self-models than fit-absent/self-models and for fit-absent/mannequins than fit-present/mannequins and fit-absent/self-models. These findings may implicate greater engagement of exploratory decision strategy and efforts to gain more information for fit-present/self-models and fit-absent/mannequins merchandise. Interestingly, the particular affinity toward self-models when fit-present and toward mannequins when fit-absent was also observed in the ACC activity, where it responded higher for fit-present/self-models than fit-present/mannequins and for fit-absent/mannequins than fit-absent/self-models and fit-present/mannequins. The rostral ACC is implicated in the computation of positive expected errors, and the dorsal ACC is involved in communicating conflict and error terms of choices (Marsh et al., 2007). As uncertainty is an inherent problem with online shopping, the evidence seems to highlight conflict when making purchasing decision for fit-present products shown on self-models and for fit-absent products displayed on mannequins. Taken together, in the context of purchasing products, participants might have engaged in a directed exploration strategy and information seeking efforts, perhaps due to a heightened sense of ambiguity regarding a given product—especially for fit-absent products on mannequins.

Our data emphasizes the fit-present/self-models and fit-absent/mannequins, where fit-present/self-models items prompted greater neural activity than fit-absent/self-models and fit-present/mannequins, and the region activity was greater toward fit-absent/mannequins than toward fit-absent/self-models. Literature regards the SFG in higher cognitive functions, and more specifically, the right SFG has been implicated in impulse and inhibitory control (Hu et al., 2016). Accordingly, such results could indicate greater demand for the cognitive control mechanism by the conditions of fit-present/self-models and fit-absent/mannequins, in particular. The greater PCC activity toward fit-present products on self-models than on mannequins could be suggesting that products with fit satisfaction displayed on the subject promotes further information processing, such as greater salience detection and greater engagements of scene and action processing (Leech and Sharp, 2014). Altogether, the findings highlight the cognitive demands and information processing of stimulus during purchase deliberation, and illustrate the influence of garment fit and product presentations on the consumer process.

Despite these meaningful results, the reward-related regions we initially expected through the hypothesis did not show activational sensitivity when deliberating purchase decisions. In particular, we expected that the interaction effect would be exhibited in the vmPFC that has been associated with high choice certainty and reward outcome (Knutson et al., 2003; Bhanji et al., 2010). However, the present task seemed to have prompted participants to make decisions with high sense of ambiguity about the product. Overall, the lack of reward-related recruitment indicates that deliberating short-lived purchase decisions based on garment fit and presentation style presented on the monitor may engage cognitive and imagery processing rather than reward processing.

### Sources of Purchase Intention

Not surprisingly, the fitness scores were higher toward intention-present products and mannequins than their respective counterparts. Such results were contrary to the results in Part 1. This perhaps reflects a common tendency, where the initial impression of an item seems more appealing when it is presented on mannequins, but in reality, the actual perception of clothing fit is subpar to the expectation.

In the univariate results, diffused neural regions responded differently depending on the decision maker’s intention-to-purchase, though no interaction was observed with product presentation; and most of these regions were resonated in MVPA as well. Most notably, the current investigation underlines the SPL function in relation to consumers’ purchase intention. The SPL is involved in mental imagery and mental rotation for body parts (Vingerhoets et al., 2002; Overney et al., 2005). The premotor cortex can also play a role in action preparation and decision commitment rather than simple motor performance (Thura and Cisek, 2014). Reflected in the longer RTs for clothing with purchase intention, the enhanced left SPL and premotor cortex for intention-present than for intention-absent conditions recommends that participants indulged a more thorough assessment of intention-present garments. Unlike the left SPL activity, the right SPL gravitated toward intention-absent garments. The lateralization of the SPL has been discussed in several studies. For example, the right is implicated in the monitoring of externally generated movements and visuospatial processing, whereas the left is suggested in the internal representation of self-generated motions and body schema (Ogawa and Inui, 2007; Sack, 2009). Together, evaluating products with subsequent purchase intention utilized more of internal processes, whereas intention-absent merchandise involved more of external processes.

The SMG is another critical structure that showed preferences toward intention-present products. The SMG contributes to decision-making under uncertainty (Vickery and Jiang, 2009) and emotion processing of fearful stimuli (Sarkheil et al., 2013), and is related to enactment effects through abundant mirror neurons (Russ et al., 2003; Chong et al., 2008). The SMG activity toward intention-present alludes that participants were envisioning themselves trying on apparel with a sense of ambiguity.

In addition to the right SPL, regions showing preferences for intention-absent clothing included the dmPFC, STS, anterior and posterior insula, and dorsal striatum. Past literature has consistently delineated the dmPFC and STS as the mentalization network, which is engaged when one makes an inference about others’ mental states, especially when other agents are similar to oneself (Frith and Frith, 2006). Similarity aids in the understanding of other’s mental states and promotes a sense of certainty (Faraji-Rad et al., 2015). Mentalization can be inferred from the dmPFC and STS activity during the evaluation of products without purchase intention. Intention-absent products encouraged participants to imagine how items would be perceived by others to achieve reassurance, denoting possible engagements of mentalization at the evaluation stage. Meanwhile, within the context of decision-making paradigm, the anterior and posterior insula have been associated with negative emotional states, aversive somatosensory integration, and risk assessment and potential loss (Paulus and Stein, 2006; Preuschoff et al., 2008; Canessa et al., 2013). This finding is in accordance with a previous intriguing study that had shown the involvement of the insula in social risk perception, which guides consumers when making purchase decisions (Yokoyama et al., 2014). Moreover, as the dorsal striatum is known to play a significant role in evaluating information of outcomes and action selection (Balleine et al., 2007; Haber, 2016), the coactivation of these structures perhaps suggests an intricate seesaw of processes that assess risk and potential loss to facilitate better action to be chosen.

Exploratory PPI analysis also underlines the involvement of mental imagery and cognitive control. Increased couplings of the SMG with the fusiform gyrus and cerebellum for intention-present than intention-absent presumably support our assertions that mental imageries of given visual input would be distinctly different when evaluating products that are later identified as having intention-to-purchase. On the other hand, as the dmPFC receives input from the sensory and parietal region to make appropriate actions (Eickhoff et al., 2016), the strengthened dmPFC-SPL for intention-absent products bolsters our above findings that illustrate an engagement of mentalization when evaluating products without intention to purchase.

Meanwhile, the product presentation effect during fit evaluation also seems to involve brain regions related to self-projection as it did during purchase deliberation. In all regions, activity increased more in self-models than in mannequins. In particular, dmPFC and TPJ responses to self-models are in line with the literature as they are prominent players for mentalization and social cognition (Saxe and Kanwisher, 2003; D’Argembeau et al., 2007). It is interesting to note that dopamine reward circuit regions such as the pOFC, nucleus accumbens, and midbrain (Ikemoto, 2007; Sescousse et al., 2010) gravitate toward self-models than mannequins, suggesting the hedonic effect of displaying items on the participants themselves.

### Comprehensive Consideration

The results of Parts 1 and 2 offer insights into the effects of garment fit, purchase intention, and product presentation on the consumer decision-making process. Part 1 showed the importance of directed exploration and visual processing that capture the interaction effects between garment fit and product presentation at choice. The garment fit effect suggested mental imagery as critical part of garment fit during purchase deliberation. Part 2 also indicated an extensive engagement of areas known for mental imagery, high population of mirror neurons, and mentalization network. Activations in regions like the SMG and SPL suggest that mental interaction with products is a critical component for both fit satisfaction and purchase intention throughout the decision process. The combinations of univariate, multivariate and exploratory PPI analyses emphasize that items without purchase intention involved participants to take into account for the perspectives of others. With respect to product presentation, both parts especially emphasized the effects of the self-model presentation. Part 1 underlined the self-projection processing evoked by self-models during purchase deliberation, whereas Part 2 accentuated the mentalization and reward processing elicited by self-models during fit evaluation. The overall findings also convey that the product presentation factor bears greater weight on the presence of garment fit but not on the presence of purchase intention. Together with the nonsignificant influence of product presentation on the prediction of subsequent purchase intention and our behavioral data from Part 1, perhaps communicates that the way products are displayed do not play a substantial role in the final decision.

Our study presented neural substrates involved in the intricate workings among factors like garment fit, product presentation methods, and purchase intention through two parts. The neural regions identified in the two parts were to reflect the categorical factors between garment fit (absence/presence) and product presentation, and between purchase intention (absence/presence) and product presentation. Furthermore, because behavioral responses were obtained on a four-point Likert scale, it would be interesting to investigate the linear modulatory effect of behavioral data using parametric modulation in future studies.

Conducting studies only in female participants and only in young adults may limit the generalizability of our interpretations, and participants’ general patterns of shopping behavior and favored styles of clothing were not accounted for. Although the price is a vital factor in shopping, this study did not allow direct examination of price effects; instead, only presented price information to control for its effects. Going forward, study designs should embrace price points to investigate how perceptions of price interact with consumer processes and product presentation types. In addition, to control for the price effect, it would have been more appropriate to include the difference between expected versus presented prices as a nuisance regressor rather than simply adjusting the prices based on each reference price. Although participants with clearly disproportionate behavioral responses between intention-to-purchase conditions were excluded, the analysis included some imbalance. Consequently, the classification accuracies of the brain regions containing intention-predictive information may have been biased by this imbalance. In similar light, the imbalance of data between fit-absent and fit-present was greater in Part 1 (effect of fit satisfaction). As such, the interpretation of the results should be read with caution.

## Conclusion

The present investigation surveyed the neural substrates of decision-making processes in online shopping for apparels. The findings of the study further the current understanding of the relationship between garment fit and purchase intention, and provide a comprehensive overview of neural correlates that characterize respective components. First, we established neural evidence for the notion that fit of clothing and modes of display have an impact on purchase decisions, suggesting that webstores should offer virtual displays of products to mitigate uncertainty and promote optimal decision strategies chosen during deliberation of purchase decision. Then the investigation discovered the neural basis of purchase intention. Reflected in the parietal lobules, prefrontal cortex, and sensorimotor-related regions, imaging data accentuated the employment of mental imageries. Collectively, the evidence featured directed exploration, visual and action processing as key factors that capture the essence of garment fit and product display during purchase deliberation, and mental interaction with products as a critical factor of fit satisfaction and purchase intention throughout the consumer process. Furthermore, the findings marked mental imagery and social cognition as predictive factors of purchase intention during fit evaluation.

## Data Availability Statement

The original contributions presented in the study are included in the article/supplementary material, further inquiries can be directed to the corresponding author/s.

## Ethics Statement

The studies involving human participants were reviewed and approved by the Institutional Review Board of Yonsei University Severance Hospital. The patients/participants provided their written informed consent to participate in this study. Written informed consent was obtained from the individual(s) for the publication of any potentially identifiable images or data included in this article.

## Author Contributions

HK and J-JK conceived and designed the experiments. HK and JK performed the experiments. HK analyzed the data and drafted the manuscript. J-JK revised the manuscript. JK edited the manuscript. All authors contributed to the article and approved the submitted version.

## Conflict of Interest

The authors declare that the research was conducted in the absence of any commercial or financial relationships that could be construed as a potential conflict of interest.

## Publisher’s Note

All claims expressed in this article are solely those of the authors and do not necessarily represent those of their affiliated organizations, or those of the publisher, the editors and the reviewers. Any product that may be evaluated in this article, or claim that may be made by its manufacturer, is not guaranteed or endorsed by the publisher.

## Footnotes

1
https://surfer.nmr.mgh.harvard.edu/optseq/

2
https://www.fil.ion.ucl.ac.uk/spm/
